# Suppression of the root-knot nematode disease through background fertilization with organic amendments

**DOI:** 10.3389/fpls.2025.1659742

**Published:** 2025-10-13

**Authors:** Paula Lillo, Sara Sánchez-Moreno, Maria Dolores Vela, Miguel de Cara-García, Miguel Talavera

**Affiliations:** ^1^ Department of Environment and Agronomy, Institute for Agricultural and Food Research and Technology, Spanish National Research Council (INIA-CSIC), Madrid, Spain; ^2^ IFAPA Rancho de la Merced, Andalusian Institute for Research and Training in Agriculture and Fisheries, Jerez de la Frontera (Cádiz), Spain; ^3^ IFAPA La Mojonera, Andalusian Institute for Research and Training in Agriculture and Fisheries, La Mojonera (Almería), Spain; ^4^ IFAPA Alameda del Obispo, Andalusian Institute for Research and Training in Agriculture and Fisheries, Córdoba, Spain

**Keywords:** cucumber, fertilizer, horticulture, *Meloidogyne*, nematode, organic amendment, soil health

## Abstract

**Introduction:**

Organic amendments like manures, sludges, and composts have significant potential to enhance soil’s physical, chemical, and microbiological conditions, aiding in the restoration of soils disturbed by intensive agricultural practices and compensating for losses due to plant pathogens. This study investigates the effects of background fertilization with organic amendments and inorganic fertilizers on the root-knot nematode (RKN) disease in cucumber and the functioning of the soil ecosystem.

**Methods:**

Field trials were conducted in a greenhouse infested with *Meloidogyne incognita*, applying six background fertilization treatments: fresh cow manure, composted cow manure, fresh chicken manure, pelletized chicken manure, slow-release inorganic fertilizer, and fast-release inorganic fertilizer. Each amendment was adjusted to provide equivalent units of N-fertilization. After 120-day crop cycles, total fruit production and RKN-disease severity were evaluated, along with nematode-based indices.

**Results:**

The fresh chicken manure treatment yielded the highest cucumber production, despite no significant differences in RKN-disease severity between treatments. Different organic amendments influenced RKN mortality at transplanting, with fresh chicken manure being the most effective in reducing RKN abundances in soil, followed by pelletized chicken manure, fresh cow manure, and composted cow manure. The inorganic fertilizers were the least effective in reducing RKN soil abundances. Organic amendments increased the complexity of the soil food web, whereas fast-release inorganic fertilizers led to its degradation and simplification. Cucumber cultivation and fertigation throughout the crop cycle enriched the soil with nutrients, intensified the bacteria-dominated organic matter degradation channel, and further simplified the soil food web.

**Discussion:**

This study demonstrates the potential of organic amendments to enhance soil health and partially suppress root-knot nematode disease in cucumber.

## 1 Introduction

Root-knot nematodes (RKNs: *Meloidogyne* spp.) are microscopic soil organisms that feed upon plant roots, causing severe damage to many crops and substantial yield losses (Jones et al., 2013). RKN-disease symptoms include root galling, poor growth, plant atrophy, loss of vigor, leaf chlorosis, wilting, and early leaf drop, leading to reduced agricultural yields. For intensive horticultural crops, control of RKN-disease has traditionally relied on reducing RKN abundances in soil before cultivation, using fumigant agrochemicals such as methyl bromide, 1,3-dichloropropene, or metam sodium. However, these fumigants have been banned or restricted in the European Union due to their adverse effects on human health and the environment. In soils with high RKN abundances (>500 J2 per 250 cm^3^ soil), nematode control measures remain necessary to achieve profitable horticultural productions (Greco et al., 2020). The most common alternatives to chemical soil fumigation include plant resistance when available, non-fumigant nematicides, and biosolarization (Sorribas et al., 2005; Ros et al., 2018; Nnamdi et al., 2022). However, in cases where RKN soil abundances are low (<500 J2 per 250 cm^3^ soil), it has been shown that the use of some chemical nematicides or biosolarization may not be cost-effective, as the application costs could surpass the benefits gained from reducing the yield losses caused by nematodes (Talavera-Rubia et al., 2022). In such cases, adequate soil and crop management are the only options to compensate for the damage caused by RKN and keep profitable horticultural yields.

Organic amendments, whether fresh, composted, or processed into pellets, can induce soil suppressiveness against the RKN-disease (Rosskopf et al., 2020). In suppressive soils, RKN establishment can occur, but RKN-disease is partially suppressed by the microbial community acting antagonistically against the pathogen or just compensating for the reduction in water and nutrient uptake caused by phytoparasitic nematodes (Sánchez-Moreno and Ferris, 2007). Moreover, several studies have shown that organic amendments improve the physical, chemical, and microbiological properties of the soil (Matisic et al., 2024). They also release essential nutrients for plants, presenting themselves as sustainable alternatives to inorganic fertilizers (Esmaeilian et al., 2022).

The type of fertilizer used in agricultural management can influence soil ecosystem services within a brief period. Organic amendments with varying C:N ratios exert multiple effects on soil functioning and food webs, enhancing soil multifunctionality (Lillo et al., 2025). On the other side, inorganic fertilizers increase plant yields and positively affect the ecosystem service of plant production when compared to organic fertilizers (Martinez et al., 2021; Deru et al., 2023). Nitrogen fertilization can promote varying effects on nematode trophic groups, thereby influencing food web dynamics and ecosystem functions such as organic matter decomposition and pest suppression (Song et al., 2016). These differences can be measured by calculating and analyzing nematode-based indices, which include i) maturity indices (Bongers, 1990) that assess nematode community condition across ecological successions, ii) food web indices (Ferris et al., 2001), developed to analyze structural characteristics of the soil food web in terms of dominant organic matter decomposition channels and complexity, and iii) metabolic footprints (Ferris, 2010), proposed to quantify the magnitude of nematode contribution to ecosystem processes. Nematode based indices have been extensively used to assess the effects of natural and anthropogenic perturbation in natural and agricultural ecosystems (Du Preez et al., 2022).

The aim of this work was to evaluate the effects of various organic amendments as background fertilizers on the RKN-disease caused by *Meloidogyne incognita* in cucumber, without previous soil disinfestation, and its implications for soil health and the functioning of the soil ecosystem. We hypothesized that background fertilization with organic amendments partially suppresses the RKN-disease in cucumber by promoting soil multifunctionality and enhancing soil health.

## 2 Materials and methods

Field trials were conducted in a 1000 m^2^ plastic greenhouse located at the Andalusian Institute of Agricultural and Fisheries Research and Training, IFAPA Chipiona experimental farm (36°44’56’’N - 6°24’06’’W). The greenhouse soil was categorized as sandy-silty (82% sand, 8% silt, 10% clay) with the following physicochemical properties: total N 1465 mg/kg, extractable Ca 370 meq/kg, extractable P 366 mg/kg, extractable Mg 30.9 meq/kg, extractable K 4.5 meq/kg, extractable Na 29.0 meq/kg, active limestone< 0.10% CaCO_3_, cation exchange capacity 106 meq/kg, carbonates 0.23% CaCO_3_, electrical conductivity at 25°C 1035 μS/cm, oxidizable organic matter 0.93%, pH at 25°C 7.8, C/N ratio 3.69, and naturally infested by *M*. *incognita* (102 ± 9 J2 per 250 cm^3^ soil).

The greenhouse soil was carefully tilled crosswise, drip irrigated until field capacity, and then eighteen plots of 28.5 m^2^ (9.5 × 3 m) were bounded. The experimental design included six types of soil background fertilization treatments, with three replicates each, randomly distributed: fresh cow manure, composted cow manure, fresh chicken manure, pelletized chicken manure, slow-release inorganic fertilizer [Floranid^®^Twin Permanent NPK 16-7-15, Compo-expert S.L. Spain], and fast-release inorganic fertilizer [Nitrofoska^®^ NPK 15-15-15, Eurochem Agro Iberia S.L. Spain] as standard background fertilization control in conventional horticultural practices (**Table 1**).

**Table 1:** Chemical properties, dosages and costs of the organic amendments and fertilizers.

Treatment	N-P-K	pH	C:N	Dosage (kg/ha)	Cost (€/ha)
Fresh Cow Manure	2.2-1.0-0.7	8.8	20:1	545	45
Composted Cow Manure	1.0-0.5-0.8	8.4	15:1	1200	100
Fresh Chicken Manure	3.3-1.6-0.8	6.8	11:1	364	75
Pelletized Chicken Manure	3.0-3.5-3.5	7.5	10:1	400	100
Slow-release inorganic fertilizer	16.0-7.0-15.0	6.5	–	75	225
Fast-release inorganic fertilizer	15.0-15.0-15.0	5.0	–	80	100

Dosages of each fertilization treatment were adjusted to deliver 159 nitrogen fertilizer units (NFU) per ha: 12 NFU were added five days before transplanting, either as organic or inorganic background fertilization treatments, and 147 NFU were supplied by fertigation during the growing season in all plots. Fertigation consisted of Structure (NPK: 7-21-0) 100 L/ha + Black up (NPK: 0-0-4) 50 L/ha twice a week (Zoberback Agrocompany S.L., Barcelona, Spain) for the first 10 weeks of cultivation, and from week 11 to the end of the crop, Diamant (NPK: 15-05-30) 6 kg/ha (Agri Nova Science S.A., Almeria, Spain) + Haifa Cal™ Calcium Nitrate (NPK: 15.5-0-0 + 26.5 CaO) 6 kg/ha (Haifa Iberia S.A., Madrid, Spain) once a week.

The trial was conducted over two winter crop cycles, from November to March, in consecutive years (2022–2023 and 2023-2024). Each plot was planted with sixteen *Cucumis sativus*, cucumber cv. Modan plants (Rijk Zwaan Ibérica S.A., Almería, Spain), arranged in two rows of eight plants, with a separation of 1 m between rows and 2 m separation between adjacent plots. Plants were irrigated as required, and weed control was performed manually throughout the growing cycle. Following 120-day crop cycles, the total fruit yield and the RKN-disease severity, estimated by root gall indices, were recorded in the six central plants of each plot (Bridge and Page, 1980).

Nematode abundances in soil were assessed at three stages of each crop cycle: before treatments (P0: one day before background fertilization treatments), at transplanting (Pi: five days after background fertilization treatments), and at harvest (Pf: 120 days after transplanting). Nematodes were extracted from subsamples of 250 cm^3^ of soil using the Whitehead tray method (Bell and Watson, 2001) and stored at 4 °C until counted and identified at a compound microscope. Counts were done on live specimens at 100× magnification using cross-linked plates. Morphotype identification was performed on approximately 100 specimens from each soil sample at 400× magnification. Identified morphotypes were assigned to trophic groups (Yeates et al., 1993), c-p, and functional guilds (Bongers et al., 1995). Nematode-based indices were calculated using the NINJA web application (Sieriebriennikov et al., 2014, https://shiny.wur.nl/ninja/).

RKN mortality and multiplication rate values were calculated for each plot by the formula:


$$RKN\;mortality=\left[1-\left(\frac{RKNPi}{RKNP0}\right)\right]\times 100$$

$$RKN\;multiplication\;rate=\left(\frac{RKNPf}{RKNPi}\right)$$

where RKNP0, RKNPi, and RKNPf are the RKN J2 abundances per 250 cm^3^ of soil at pre-treatment, transplanting, and harvest, respectively.

All results are expressed as mean ± standard error of the mean. Statistix v.9.0 software (Analytical Software, Tallahassee, FL, USA) was used to analyze the data. Data were subjected to the Shapiro-Wilk and Brown-Forsythe tests to determine whether the variances were normal and homoscedastic. If significant, data were *logx* or *√x* transformed and evaluated again. When normality and homoscedasticity of variances could be assumed, data were analyzed using ANOVA. The HSD Tukey’s test (p<0.05) was used to compare the means if the F values were significant. When the homoscedasticity of variances could not be assumed, Welch’s ANOVA was used. Kruskal-Wallis non-parametric tests were used to analyze the data when normality was not attained following transformation. Dunn’s multiple comparison test was used to compare medians if H values were significant (p<0.05). If no significant effects of the cropping cycle were found, data from both cycles (2022–2023 and 2023-2024) were combined and treated as repeated measures of the same background fertilization treatment.

## 3 Results

### 3.1 Effects of organic amendments on RKN-disease symptoms and RKN soil populations

**Table 2** shows cucumber yield and RKN-disease data from field trials conducted in a greenhouse infested by *M*. *incognita* without previous soil disinfestation. Distribution of RKN abundances was homogenous between plots at the pretreatment sampling time (P0). Different organic amendments influenced the RKN mortality at transplanting (Pi), the fresh chicken manure being the most effective in reducing RKN abundances in soil (p<0.05), followed by pelletized chicken manure, fresh cow manure, and composted cow manure (**Table 2**). The inorganic fertilizers were the least effective in reducing RKN soil abundances. There were no effects of the different background fertilization treatments on RKN multiplication rates at harvest (Pf) (p>0.05). No significant differences between treatments were observed in the RKN-disease severity, estimated by root gall indices at harvest, but the fresh chicken manure yielded higher cucumber production (p<0.05) than the rest of the treatments (**Table 2**).

**Table 2:** Cucumber yield per plant, RKN galling indices, initial RKN soil densities, RKN mortality after soil fertilization treatments, and RKN multiplication rates at harvest (Pf/Pi) in two field trials with six organic amendments and N-fertilization treatments.

Treatment	RKN abundances at P0*	RKN mortality at Pi (%)*	RKN multiplication at Pf*	RKN gall index at Pf**	Fruit yield (kg/plant)**
Fresh Cow Manure	90.67 ± 9.56 a	30.95 ± 2.99 bc	4.82 ± 1.29 a	5.03 ± 0.28 a	7.09 ± 0.10 b
Composted Cow Manure	110.33 ± 9.80 a	24.86 ± 2.34 c	4.72 ± 0.70 a	4.83 ± 0.22 a	6.50 ± 0.58 b
Fresh Chicken Manure	167.50 ± 30.84 a	47.37 ± 2.02 a	5.20 ± 1.06 a	5.19 ± 0.27 a	8.26 ± 0.24 a
Pelletized Chicken Manure	120.50 ± 21.40 a	37.31 ± 1.98 b	5.32 ± 1.32 a	4.72 ± 0.29 a	6.92 ± 0.25 b
Slow-release inorganic fertilizer	111.33 ± 10.88 a	11.74 ± 1.47 d	3.33 ± 1.31 a	4.86 ± 0.27 a	5.87 ± 0.53 b
Fast-release inorganic fertilizer	97.83 ± 7.98 a	9.78 ± 1.57 d	4.25 ± 0.78 a	4.69 ± 0.19 a	5.45 ± 0.17 b

*RKN abundances, mortality, and multiplication factor data are the mean ± standard error of six replicates (three plots × two cropping cycles).

**Cucumber yield and RKN gall index data are the mean ± standard error of thirty-six replicates (six plants × three plots × two cropping cycles).

Values within the same column followed by the same letter do not differ significantly (p<0.05) according to ANOVA-Tukey or Kruskal-Wallis-Dunn tests.

### 3.2 Effects of organic amendments and inorganic N-fertilizers on nematode community structure and soil food web

#### 3.2.1 Total nematode abundances

There were no differences in the number of nematodes, their biomass, or any of the ecological indices measured between cropping cycles, but the basal index (BI), an indicator of depleted soil food webs (Du Preez et al., 2022), and the enrichment footprint (EF), an indicator of organic enrichment and soil fertility (Du Preez et al., 2022), varied across cropping cycles. The BI was lower in 2023-2024 (32.25 ± 1.58) than in the 2022–2023 cropping cycle (36.23 ± 1.10) (p<0.05) and the EF was lower in 2022-2023 (37.18 ± 4.14) than in the 2023-2024 (48.428 ± 5.78) cycle (p<0.05), indicating a general nutrient enrichment in soils from 2022 to 2024.

#### 3.2.2 Nematodes based indices

**Table 3** illustrates the variation of different nematode based indices across various sampling times, without considering the effects of the different background fertilization treatments. Overall, total nematode abundance and biomass were maintained or reduced from P0 to Pi but increased from Pi to Pf (p<0.05).

**Table 3:** Effect of background N-fertilization on abundances, biomass and nematode-based indices at three sampling times (P0: before fertilization, Pi: after background fertilization, planting, Pf: after harvest of a cucumber crop).

Indices	P0		Pi		Pf	
Abundance (nematodes·250 cm^-3^ soil)	1086.10 ± 71.94	ab	943.39 ± 55.82	b	1306.50 ± 98.43	a
Biomass (μg·250 cm^-3^ soil)	11.32 ± 0.71	b	8.40 ± 0.41	c	31.12 ± 2.69	a
Maturity index (MI)	2.18 ± 0.02	a	2.28 ± 0.04	a	1.71 ± 0.02	b
Maturity 2–5 index (MI 2-5)	2.34 ± 0.02	a	2.37 ± 0.04	a	2.11 ± 0.02	b
Sigma maturity index (∑MI)	2.59 ± 0.01	a	2.62 ± 0.02	a	2.24 ± 0.02	b
Plant parasitic index (PPIs)	2.95 ± 0.00	b	2.95 ± 0.01	ab	2.97 ± 0.00	a
Channel index (CI)	36.54 ± 1.23	b	56.72 ± 3.66	a	9.70 ± 0.89	c
Basal index (BI)	35.43 ± 0*.7*4	a	39.25 ± 1.80	a	28.04 ± 1.76	b
Enrichment index (EI)	50.41 ± 0.86	b	41.88 ± 1.87	c	70.10 ± 1.86	a
Structure index (SI)	44.15 ± 1.37	a	44.53 ± 2.75	a	17.09 ± 2.30	b
Compound metabolic footprint (CF)	1524.90 ± 93.23	b	1156.70 ± 54.21	b	3948.80 ± 337.90	a
Enrichment metabolic footprint (EF)	27.89 ± 2.10	b	19.80 ± 1.67	b	80.73 ± 6.91	a
Structure metabolic footprint (SF)	145.08 ± 11.46	a	152.95 ± 18.73	a	47.80 ± 7.64	b
Bacterivorous metabolic footprint (BF)	75.98 ± 5.31	b	64.21 ± 5.40	b	138.59 ± 11.38	a
Fungivorous metabolic footprint (FF)	11.03 ± 0.93	a	11.10 ± 1.25	a	10.27 ± 0.97	a
Herbivorous metabolic footprint (HF)	1293.20 ± 84.57	b	928.73 ± 49.67	b	3752.60 ± 326.74	a
Omnivorous metabolic footprint (OF)	144.64 ± 11.41	a	152.65 ± 18.70	a	47.27 ± 7.59	b

Data are the mean ± standard error of six replicates (three plots × two cropping cycles).

Values within the same row followed by the same letter do not differ significantly (p<0.05) according to ANOVA-Tukey or Kruskal-Wallis-Dunn tests.

The maturity indices (MI, MI 2-5, ∑MI), indicators of soil food web succession (Du Preez et al., 2022), were maintained from P0 to Pi, then reduced from Pi to Pf (p<0.05) (**Table 3**). Conversely, there was an increase in the plant parasitic index (PPI), a maturity index applied to the plant-parasitic assemblage, from P0 to Pf (**Table 3**).

The channel index (CI), an indicator of fungal-mediated decomposition channel (Du Preez et al., 2022), the basal index (BI), and the structure index (SI), indicators of soil food web complexity (Du Preez et al., 2022), were slightly increased or maintained from P0 to Pi, but reduced from Pi to Pf (p<0.05) (**Table 3**). Conversely, the enrichment index (EI) was reduced from P0 to Pi and then increased from Pi to Pf (p<0.05) (**Table 3**).

Finally, our results on nematode metabolic footprints, indicators of the magnitude of the function developed by different nematode functional guilds (Du Preez et al., 2022), showed that the composite footprint (CF), which included the whole community, the enrichment footprint, which accounts for bacterial-feeding enrichment indicators (EF), the bacterivore footprint (BF), which includes all bacterivores, and herbivore footprints (HF), which considers all plant parasites, were maintained from P0 to Pi, but increased from Pi to Pf (p<0.05) (**Table 3**). The structure footprint (SF) and omnivore footprint (OF), which look at nematodes in all high trophic groups and omnivores, did not change from P0 to Pi but decreased from Pi to Pf (p<0.05) (**Table 3**). We found no differences in the fungivore footprint (FF) at any sampling time.

**Figure 1:**
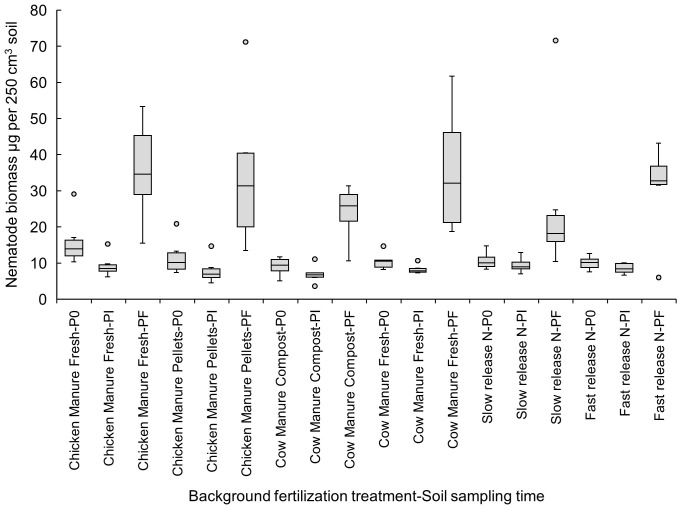
Effects of six background fertilization treatments on total nematode biomass in soil (µg per 250 cm^3^ of soil) at three sampling times through a cucumber crop: P0 pre-treatment, Pi transplanting, and Pf harvest.

When comparing the effects of the different background fertilization treatments at the three sampling times during the crop cycle, the increase in nematode biomass from Pi to Pf was significant (p<0.05) only in the following organic amendment treatments: pelletized chicken manure (Pi: 7.93 ± 1.47 vs. Pf: 34.49 ± 8.64 µg per 250 cm^3^ of soil), fresh cow manure (Pi: 8.29 ± 0.52 vs. Pf: 35.51 ± 7.13 µg per 250 cm^3^ of soil), and composted cow manure (Pi: 6.91 ± 1.00 vs. Pf: 23.95 ± 3.10 µg per 250 cm³ of soil) (**Figure 1**).

**Figure 2:**
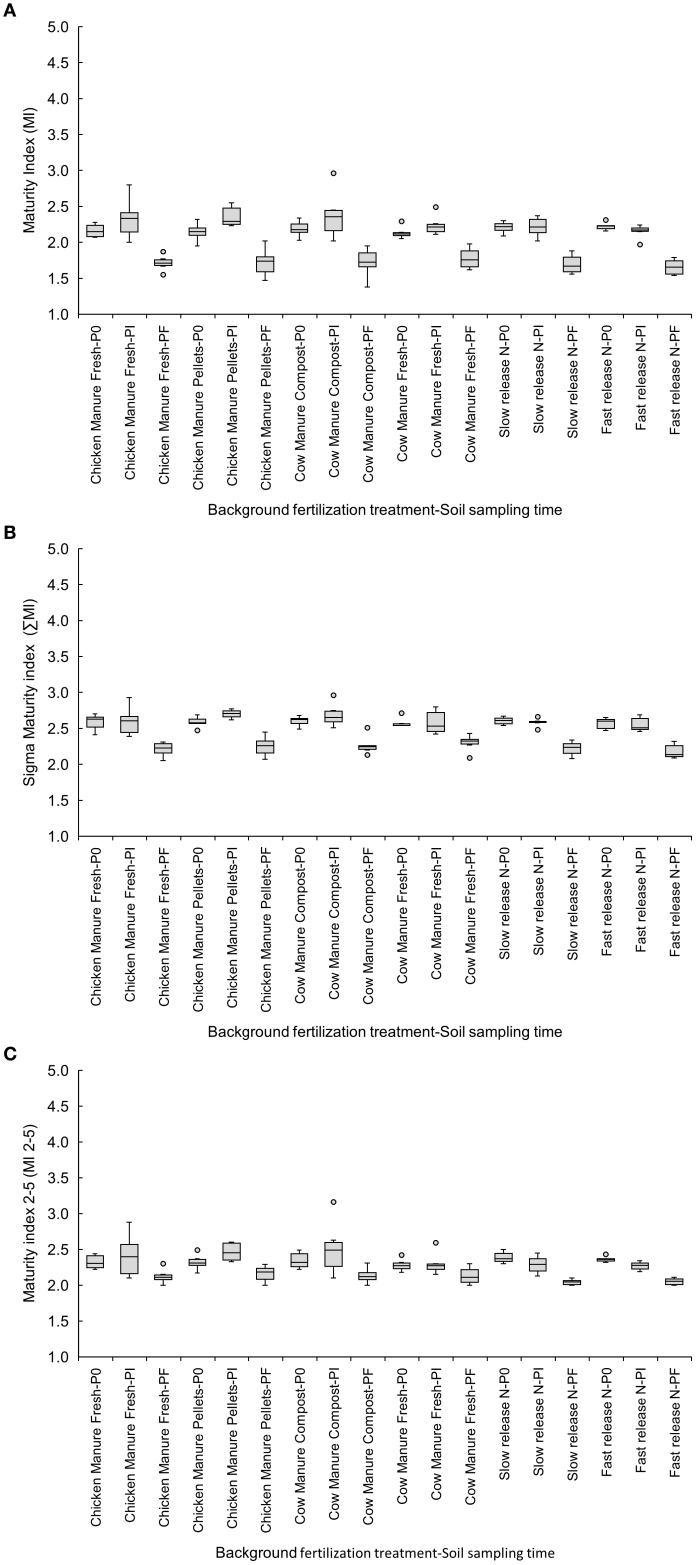
Effects of six background fertilization treatments on maturity indices MI **(A)**, ∑MI **(B)**, and MI 2-5 **(C)** at three sampling times through a cucumber crop: P0 pre-treatment, Pi transplanting, and Pf harvest.

The maturity indices decreased from Pi to Pf in the pelletized chicken manure, with MI showing values of 2.36 ± 0.06 for Pi and 1.72 ± 0.08 for Pf (**Figure 2A**), and ∑MI showing 2.70 ± 0.02 for Pi and 2.25 ± 0.06 for Pf (**Figure 2B**). MI 2–5 decreased from P0 to Pi in both the fast-release (P0: 2.36 ± 0.02 vs. Pi: 2.27 ± 0.03) and slow-release (P0: 2.39 ± 0.03 vs. Pi: 2.29 ± 0.05) inorganic fertilizers (**Figure 2C**).

**Figure 3:**
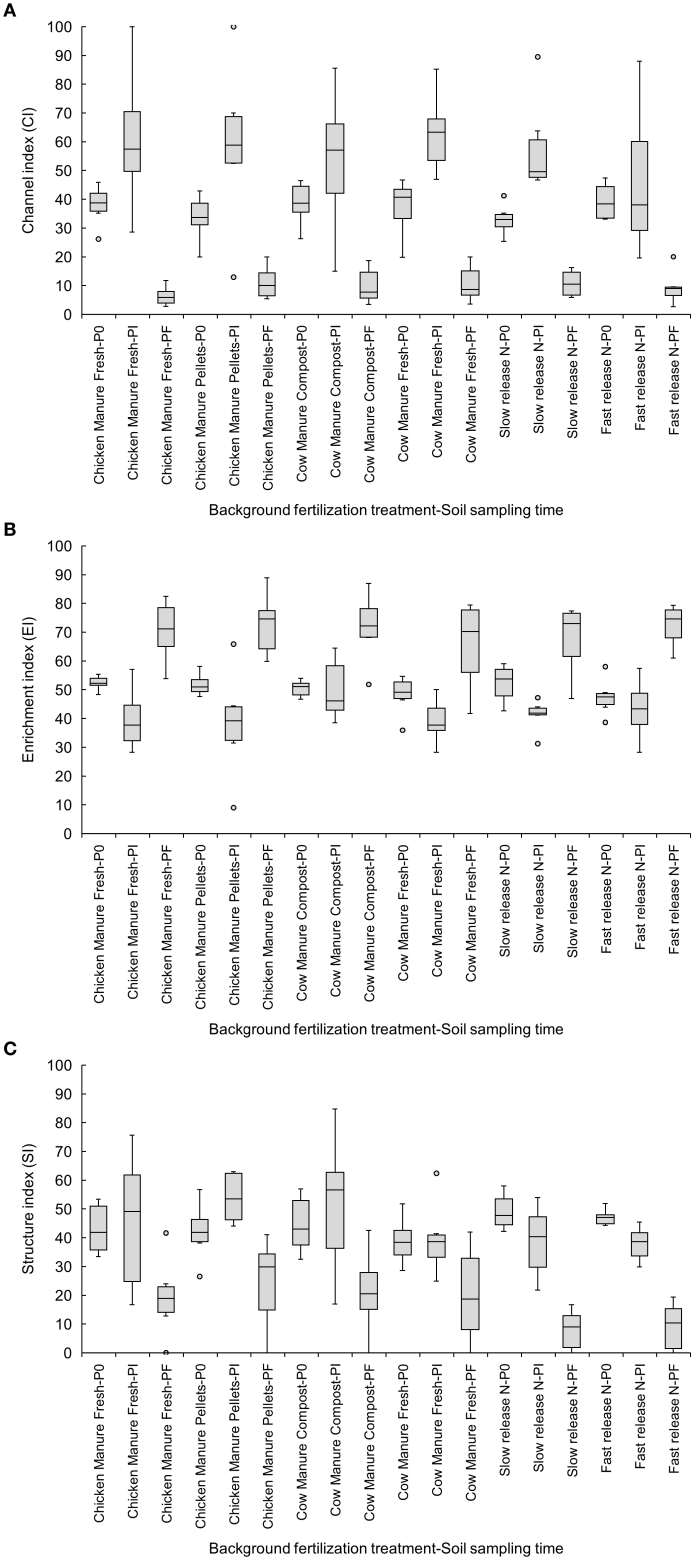
Effects of six background fertilization treatments on channel **(A)**, enrichment **(B)**, and structure **(C)** indices at three sampling times through a cucumber crop: P0 pre-treatment, Pi transplanting, and Pf harvest.

All background fertilization treatments showed a trend to reduce CI from Pi to Pf, but these reductions were statistically significant (p<0.05) only in the slow-release inorganic fertilizer (Pi: 57.76 ± 6.85 vs. Pf: 10.76 ± 1.86), fresh chicken manure (Pi: 60.89 ± 9.90 vs. Pf: 6.40 ± 1.36), and fresh cow manure (Pi: 63.08 ± 5.63 vs. Pf: 10.70 ± 2.62) (**Figure 3A**). The increase in the EI from Pi to Pf was significant (p<0.05) only in chicken manure treatments, fresh (Pi: 39.68 ± 4.34 vs. Pf: 70.46 ± 4.36) or pelletized (Pi: 38.19 ± 7.60 vs. Pf: 72.88 ± 4.48) (**Figure 3B**). The SI was reduced significantly (p<0.05) from P0 to Pf in both inorganic fertilizers, fast- (P0: 47.11 ± 1.17 vs. Pf: 9.25 ± 3.43) and slow-release (P0: 49.11 ± 2.54 vs. Pf: 8.05 ± 2.84) (**Figure 3C**).

**Figure 4:**
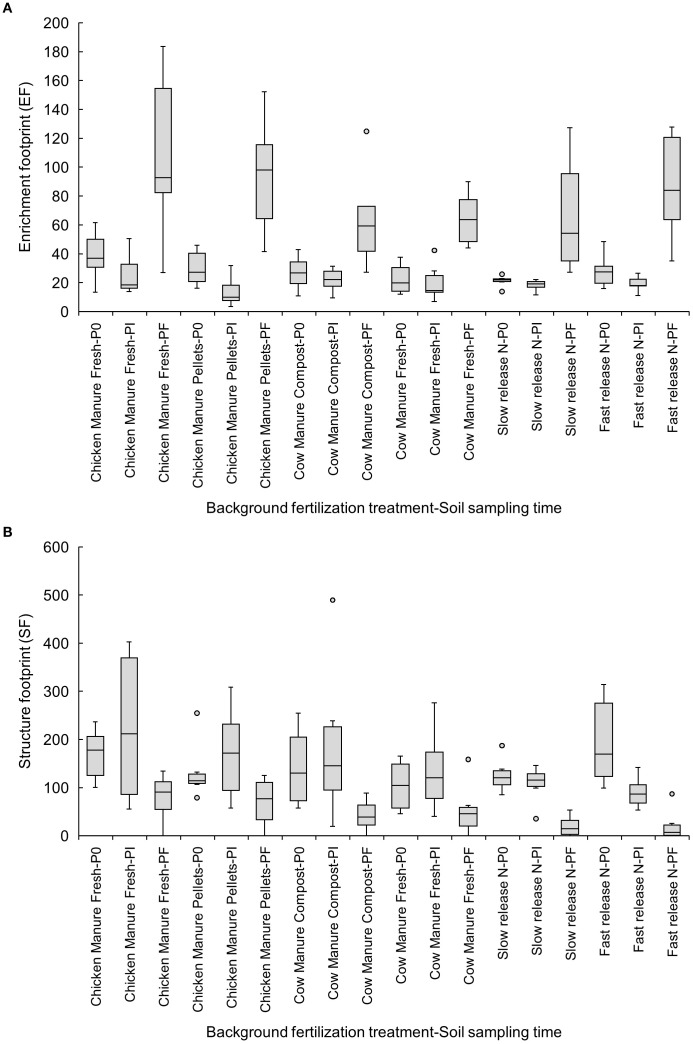
Effects of six background fertilization treatments on enrichment **(A)** and structure footprints **(B)** at three sampling times through a cucumber crop: P0 pre-treatment, Pi transplanting, and Pf harvest.

The EF increased at Pf in all treatments except fresh or composted cow manure (**Figure 4A**). Fast- (P0: 195.12 ± 38.34 vs. Pf: 21.17 ± 13.74) and slow-release inorganic fertilizers (P0: 125.41 ± 14.71 vs. Pf: 19.69 ± 8.70) reduced the SF from P0 to Pf (**Figure 4B**).

**Figure 5:**
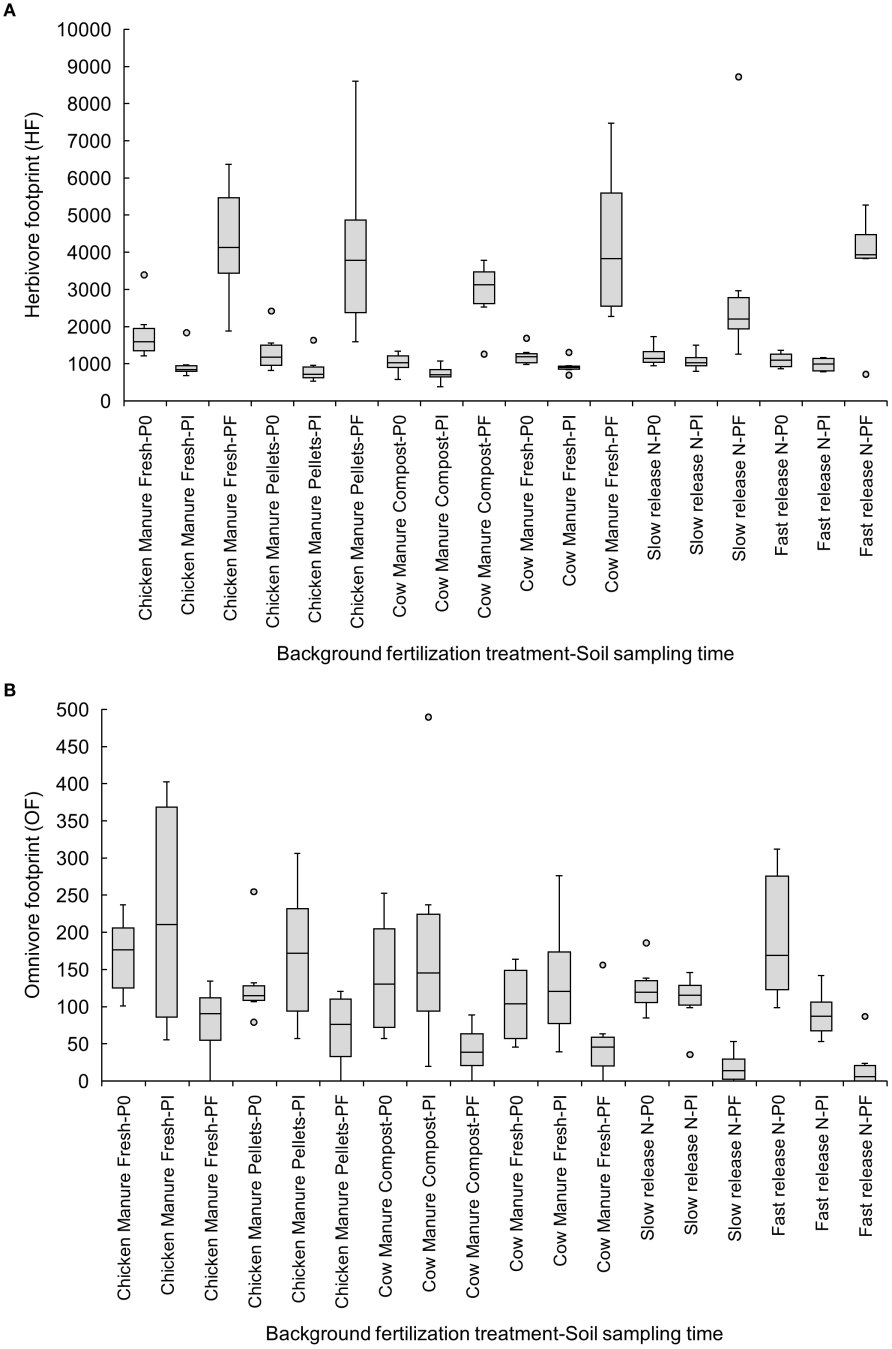
Effects of six background fertilization treatments on herbivore **(A)** and omnivore footprints **(B)** at three sampling times through a cucumber crop: P0 pre-treatment, Pi transplanting, and Pf harvest.

Herbivore footprints increased from Pi to Pf in all organic amendments, but not in inorganic fertilizers (**Figure 5A**). The omnivore footprint decreased from P0 to Pi in the fast-release inorganic fertilizer (P0: 194.43 ± 38.19 vs. Pf: 20.40 ± 13.84) (**Figure 5B**).

## 4 Discussion

### 4.1 Organic amendments promote RKN-disease suppression

#### 4.1.1 RKN disease suppression by organic amendments in cucumber crops

Our study demonstrates the potential of organic amendments to partially suppress RKN-disease in cucumber. At the pre-treatment stage (P0), RKN abundances were uniformly distributed across the field plots. At the transplanting stage (Pi), five days after the background fertilization treatments, RKN soil abundances (83 ± 27 J2 per 250 cm^3^ of soil) exceeded the tolerance limits estimated for the *C*. *sativus*-*M*. *incognita* pathosystem (0.13-0.25 J2 per 250 cm^3^ of soil) (Giné et al., 2014; Talavera-Rubia et al., 2022), and hence some yield losses were expected. Average cucumber yields in our field trials ranged from 5.45 to 8.26 kg per plant. The fast-release inorganic fertilization treatment, the standard for intensive horticultural production in the area, gave the lowest yield value. In comparison with the fresh chicken manure treatment, the fast-release N inorganic fertilization treatment was 44% less productive.

Previous research found that the application of chicken manure to soil at high dosages suppressed RKN-disease in numerous crops worldwide by releasing compounds like ammonia, organic acids, and phenolics during decomposition, which are toxic to nematodes (Peiris et al., 2020). The reported quantity of organic amendments required for effective disease control (20000–50000 kg/ha) (Melero-Vara et al., 2012) is often not affordable in many cropping systems, prompting the exploration of various approaches to optimize their effects. Our research demonstrates that when RKN soil inocula is not very high (<102 ± 9 J2 per 250 cm^3^ soil), even low dosages of organic amendments (400–1200 kg/ha) can achieve effective and profitable control of the RKN disease in highly susceptible crops such as cucumber and likely in less susceptible crops like tomato or pepper. Effectiveness of low dosages of organic amendments when RKN soil inoculum is higher or in spring-summer cropping cycles, when RKN activity is higher and damage threshold lower, still needs to be investigated.

#### 4.1.2 Mechanisms of RKN-disease suppression by organic amendments

The mechanisms of RKN-disease suppression by organic amendments can be direct, involving the release of antibiotics, toxic metabolites, or volatile organic compounds (VOCs) that are harmful to nematodes, or indirect, by enhancing populations of saprophytic or antagonist organisms in soil, boosting plant resistance to nematodes, or improving plant nutritional status, thereby increasing plant vigor to overcome the impacts of RKN (Melero-Vara et al., 2012; Rosskopf et al., 2020). Organic amendments with a higher N content (a lower C:N ratio) and easily oxidizable C have been found to produce higher concentrations of organic acids, thereby enhancing soil disinfestation efficacy (Liu et al., 2016). Nevertheless, organic amendments with high N concentration may lead to an undesired flash release of nutrients. Liu et al. (2016) highlighted the need to choose the right organic amendments and application rates that can control soilborne diseases without causing harm to the environment. In our study, the most effective organic amendments for reducing RKN abundances were those with the lowest C:N ratio; specifically, fresh and pelletized chicken manure resulted in RKN mortalities of 47.37 ± 2.02 and 37.31 ± 1.98, respectively, which aligns with previous reports indicating that higher N content improves pathogen suppressiveness (Liu et al., 2016). Additionally, chicken or chicken manure enhances soil structure, increases microbial activity, and boosts enzyme functions such as phosphatases and urease, which are essential for nutrient cycling and plant resilience. It also improves cucumber plant performance in soil infested with RKN, further explaining the higher cucumber yield observed in the fresh chicken manure treatment (Mardani et al., 2024).

#### 4.1.3 Profitability of using organic amendments in RKN-disease suppression

The Seinhorst models indicate that for the *C. sativus*–*M. incognita* pathosystem, if a nematicidal treatment costs between 50 and 100 €/ha, the RKN abundances at which the cost of treatment equals the loss in crop value (economic threshold) will be around 5–10 J2 per 250 cm^3^ of soil (Talavera-Rubia et al., 2022). If RKN abundance is higher than the economic threshold, using nematicidal treatments makes financial sense because the extra money earned from reducing yield losses will be greater than the cost of the treatment. Therefore, since the efficacies of organic amendments in reducing RKN abundances in soil ranged from 25 to 47%, their use would be profitable for RKN abundances over P0: 7–15 J2 per 250 cm^3^ of soil. Organic amendments were better at killing nematodes than inorganic fertilizers (10–12%), but not as good as most chemical nematicides allowed in the European Union (40–64%); however, organic amendments are usually free or inexpensive if obtained locally, and even when considering transportation and application costs, they were cheaper (45–100 €/ha) than the cost of approved chemical nematicide treatments in the area (170–849 €/ha) (Talavera-Rubia et al., 2022).

### 4.2 Organic amendments promote soil food web enrichment and soil multifunctionality

#### 4.2.1 Effects of organic amendments on nematodes abundances

Our results show that both organic amendments and N fertilizers exert a significant effect on the soil nematode community. Soil nematodes exhibit increased abundances and total biomass in response to N-fertilization, mainly due to increased abundances of nematodes in lower trophic levels (Okada and Harada, 2007; Liu et al., 2016), which, in turn, promotes N mineralization and increases mineral N contents in the soil (Buchan et al., 2013). However, significant increases in nematode abundances were only noted in our trials at harvest, following cucumber cultivation cycles. Even though N fertilizers might have toxic effects on soil organisms in the short term (Sachdeva et al., 2025), no negative effects of organic or inorganic fertilization on total nematode abundances were detected five days after background fertilization treatments, in agreement with previous studies (Martinez et al., 2021). Nonetheless, a positive effect of fertilization on total nematode abundance was noted at harvest, after one crop cycle of cucumber, due to the initial low resource availability in the sandy-silty soil, the fertigation applied during the crop cycle, and the presence and activity of roots.

#### 4.2.2 Effects of organic amendments on maturity indices

Maturity indices values (MI, MI 2-5, ∑MI) indicated significant soil disturbance with low food web structure at P0 and Pi, probably due to tillage and nutrient enrichment (Puissant et al., 2021), which further declined by Pf due to harmful effects of fertilizers in high soil food web levels and the promotion of lower guilds (Liu et al., 2016) from the enrichment caused by fertigation. Conversely, the plant parasitic nematode index (PPI) slightly increased post-cropping at Pf, driven by the increase in RKN nematode abundances at the end of the crop. Fresh and pelletized chicken manure reduced MI and ∑MI indices while increasing compound, enrichment, and herbivore metabolic footprints from Pi to Pf. In contrast, fast-release inorganic fertilizer reduced the structure index (SI) and MI2–5 from P0 to Pf. Therefore, the use of organic amendments or slow-release inorganic fertilizers as background fertilization maintained a more complex and structured soil food web than under a fast-release inorganic fertilizer treatment.

#### 4.2.3 Effects of organic amendments on soil food web indices

Before application of fertilization treatments at P0, soil food web indices (BI, CI, EI, and SI) exhibited low to intermediate values, indicative of moderate nutrient enrichment and a food web of low-intermediate complexity, where organic matter decomposition is predominantly carried out by bacteria, which is typical of agricultural soils with high nutrient resources (Ugarte et al., 2013). Organic amendments increased BI and CI at transplanting (Pi) due to the increase in generalist nematodes adapted to perturbation, but these indices were reduced across all treatments at harvest (Pf), while the EI showed an opposite pattern, decreasing from P0 to Pi and greatly increasing at Pf as a response to the mineralization of organic matter that boosts microbial communities, which, in turn, enhance enrichment-opportunistic nematodes (Leroy et al., 2009). The SI, an indicator of soil food web complexity, remained at intermediate values between P0 and Pi but was reduced at Pf, as commonly found in response to soil perturbation associated with agricultural management (Ferris et al., 2001). These findings indicate that although adding organic materials improved the soil food web’s complexity when plants were transplanted, this improvement decreased by the time of harvest, pointing to a change towards simpler soil food webs where bacteria break down organic matter because of ongoing nutrient supply from fertigation.

#### 4.2.4 Effects of organic amendments on metabolic footprints

Metabolic footprints, which indicate nematode contribution to ecosystem services, showed no differences between P0 and Pi, suggesting that background fertilization treatments did not affect soil food web functioning in the 5-day period after application. However, after a crop cycle, compound, enrichment, bacterivore, and herbivore metabolic footprints increased at Pf, reflecting higher nematode abundances, biomass, and contribution to soil food web carbon flux through the bacterivore and herbivore channels at the end of the cropping cycle. Conversely, structure, fungivore, and omnivore metabolic footprints decreased at Pf, indicating reduced contribution of nematodes to the fungi-mediated organic matter decomposition and low participation in ecosystem services derived from soil food web complexity, e.g. pest control (Sánchez-Moreno and Ferris, 2007; Timper et al., 2012).

#### 4.2.5 Effects of organic amendments on soil nematode community structure

Our findings indicate that when more carbon (C) is added through fertilizers, the total number of nematodes increases. However, using more nitrogen (N) from fertilizers reduces the maturity index (MI) and the number of omnivore-predator nematodes, which means that high nitrogen levels make the nematode community less complex, as noted before (Sánchez-Moreno and Ferris, 2007). Furthermore, organic amendments differ in their impact on soil nematodes, as those with C-rich crop residues supported larger free-living nematode populations and promoted structure and enrichment index (EI), whereas N-rich animal manure was more effective in controlling plant-feeding nematodes (Liu et al., 2016).

## 5 Conclusions

Our research demonstrates that low dosages of organic amendments provide effective and profitable control of RKN in highly susceptible crops such as cucumber without a previous soil disinfestation treatment and can be an alternative method to control plant parasitic nematodes when soil infestations are low.

The impacts of organic amendments on the soil nematode community (as inferred by maturity indices), soil food web structure (as assessed by soil food web indices), and ecosystem services (as inferred by metabolic footprints) were noticeable. If we assess soil health as a combination of biological indicators based on nematode fauna, we have found that the starting point at the beginning of the cropping cycle (P0) is characterized by altered, N-enriched soils, as often occurs in soils dedicated to intensive horticultural cultivation. After the application of organic amendments, an increase in the complexity of the food web was observed, while, oppositely, the addition of fast-release inorganic

fertilizers implies a quick degradation and simplification of the food web that remains depleted to the end of cucumber cultivation. Besides, the continuous fertigation throughout the cropping season caused an enrichment of the soil in nutrients, with an increase in the organic matter decomposition channel dominated by bacteria and a simplification of the food web that was maintained until the end of the crop.

In summary, our results show that organic amendments not only improve soil health but also offer a sustainable alternative to chemical fertilizers. These findings align with global efforts to promote sustainable agriculture by enhancing soil biodiversity and ecosystem services.

## Data Availability

Publicly available datasets were analyzed in this study. This data can be found here: https://doi.org/10.6084/m9.figshare.29478395.
